# The social determinants of health of the Urak Lawoi’ of southern Thailand

**DOI:** 10.1186/s12889-020-8283-y

**Published:** 2020-02-06

**Authors:** Maura Reap, Samittra Pornwattanavate, Charlie Thame, Marc Van der Putten

**Affiliations:** 1 School of Global Studies, Thammasat University, Rangsit, Thailand; 2 Faculty of Public Health, Thammasat University, Rangsit, Thailand; 3 Faculty of Political Science, Thammasat University, Bangkok, Thailand; 4 Center of Excellence in Global Health, Faculty of Public Health, Thammasat University, Rangsit, Thailand

**Keywords:** Social determinants of health, Human rights, Inequities, Marginalized, Indigenous, Health, Policy, Law, Land grabbing, Thailand, Urak Lawoi’

## Abstract

**Background:**

Traditionally, most Western models of health viewed sickness and disease as a product of individual factors such as personal behaviors and genetic predisposition; consequently, healthcare interventions were largely focused on fixing the individual, with little attention placed on contributing external factors. The WHO’s “Social Determinants of Health” (SDH) framework, however, takes a broader ecological perspective that suggests that interventions must occur at multiple levels in order for good health to be achieved on an equitable basis. This model views health as a function of many circumstantial and environmental factors that are continuously and simultaneously interacting across multiple domains. These factors include structural mechanisms, such as laws and policies; socio-economic conditions, such as education and occupation; and intermediary circumstances, such as living and working conditions. Utilizing the SDH framework as a guide, this qualitative study sought to identify which specific determinants are most significant and present the greatest risk to the health and well-being of the Urak Lawoi’ (UL), a “sea nomad” group indigenous to southern Thailand.

**Methods:**

Interviews, household surveys, and focus group discussions were utilized to gather primary data from 71 subjects in three different UL communities in southern Thailand. In addition, a comprehensive literature review of relevant international mechanisms, national laws, and national policies was conducted. All data collected was analyzed and coded utilizing HyperRESEARCH.

**Results:**

In all three communities, education and livelihoods were found to be the most critical determinants. Additionally, land grabbing and living conditions were identified as dire issues on Ko Lipe. The law and policy review revealed several deviations between international mechanisms and national laws and policies in both enshrinement and enforcement, with the Royal Thai Government (RTG) often overlooking the interests of the UL when formulating laws and policies.

**Conclusions:**

The above-mentioned determinants, along other structural and intermediary determinants, are synergizing, thereby placing the UL at increased risk of poorer health and health outcomes compared to other Thais living in the same vicinities. To rectify this, the RTG must reform national laws and policies that harm the UL, and civil society must hold them accountable. Several recommendations are offered to achieve a better future for the Urak Lawoi’.

## Background

According to the World Health Organization (WHO), disparities in health and well-being are attributable to the specific circumstances in which “people are born, grow, live, work, and age”. Collectively, these circumstances are known as the “Social Determinants of Health” (SDH), and include laws, policies, economics, livelihood, education, living conditions, etc. Within each society, those who hold the power and resources determine which specific circumstances are to be valued and which are not. Most typically, the decisions made by power yielders protect the values, interests, and well-being of themselves and their constituents. Meanwhile, the values and interests of marginalized populations tend to suffer across various domains, and this compromises their health. Thus, personal health is not solely influenced by factors such as genetics and lifestyle, but also by systemic societal inequities [1].

Indigenous peoples represent a particularly salient example of how social inequities in tandem with other SDH can negatively impact health. In 2009, the United Nations (UN) released a comprehensive analysis paper entitled “The State of the World’s Indigenous People” [2]. The findings of this analysis clearly illustrated that the socio-economic status of indigenous populations in nations and territories around the world is substantially lower than that of others living within the same area. Collectively, indigenous people are less educated than their non-indigenous neighbors and have fewer decent livelihood opportunities available to them. They are more likely to live in poverty, and they are more vulnerable to human rights infringements such as land grabbing. Disparities also extend into health and well-being, with indigenous peoples experiencing higher rates of disability, shorter lifespans, and poorer health outcomes; in some countries, the life expectancy of indigenous peoples is 20 years less than that of non-indigenous groups living in the same area [2].

This study explored the SDH of the Urak Lawoi’ (UL), a “sea nomad” people indigenous to southern Thailand whose socio-economic status is significantly lower than that of their ethnic Thai neighbors. A review of the literature reflects that the UL have consistently been subjected to on-going systemic discrimination in schools, public healthcare settings, and the justice system for well over 100 years [3]. The Thai education is monolingual and exclusively reflects the cultural values of the dominant population; thus, it fails to teach the UL the skills needed to support their traditional roles as fishermen [3, 4]. Healthcare services, also, are not sensitized about UL concepts of health, and providers are rarely familiar with the UL language even on a functional level. In government matters, discrimination, coercion, and corruption has frequently infringed upon the rights of the UL [3]. As a result, the UL have remained disenfranchised from government services that should be improving their quality of life along with everyone else’s.

Thailand does not disaggregate the data it collects according to ethnicity; it is therefore impossible to provide statistical evidence that conclusively demonstrates that the health of UL is compromised compared to others. However, deducing from the WHO SDH framework in tandem with the UN’s 2009 analysis, it is highly likely that they - like most other indigenous groups - suffer from poorer health and health outcomes than their ethnic Thai neighbors. If this is true, the only way to reverse this trend is to identify and then address the specific determinants that have contributed to and perpetuate such adverse outcomes.

To this end, utilizing the WHO SDH framework as a theoretical guide, this study analyzed several structural and intermediary determinants to better understand which present the most significant threats to UL health and well-being. This researcher hopes that the information gleaned from this research will help provoke meaningful policy dialogue at the government level, while providing civil society with the information necessary to gain traction on this critical human rights issue. In addition, the study’s findings will also add to the growing compendium of evidence that demonstrates the critical role of the SDH upon the health and wellbeing of specific vulnerable populations such as indigenous peoples.

### The WHO SDH framework

In the past, health was perceived dichotomously; people were either sick and in need of treatment, or they were healthy and did not. Personal health was typically attributed to factors such as genetics and behaviors, and this ultimately placed the burden of good health on the individual. Health promotion initiatives and interventions were designed accordingly and focused on the prevention and treatment of diseases at the individual level. However, over the past few decades, various ecological frameworks that consider health within a larger context have been gaining momentum.

Among the most prominent of the alternative frameworks is the World Health Organization’s (WHO) Social Determinants of Health (SDH) framework [1], which considers health as a function of both upstream and downstream factors and processes - known as the “Social Determinants of Health” (SDH) - that are continuously interacting in dynamic and non-linear ways over the course of an individual’s lifetime. In other words, the SDH are the social, physical, and economic circumstances in which people “are born, grow, live, work, and age”. The WHO framework [1] below illustrates how this unfolds:

**Fig. 1:**
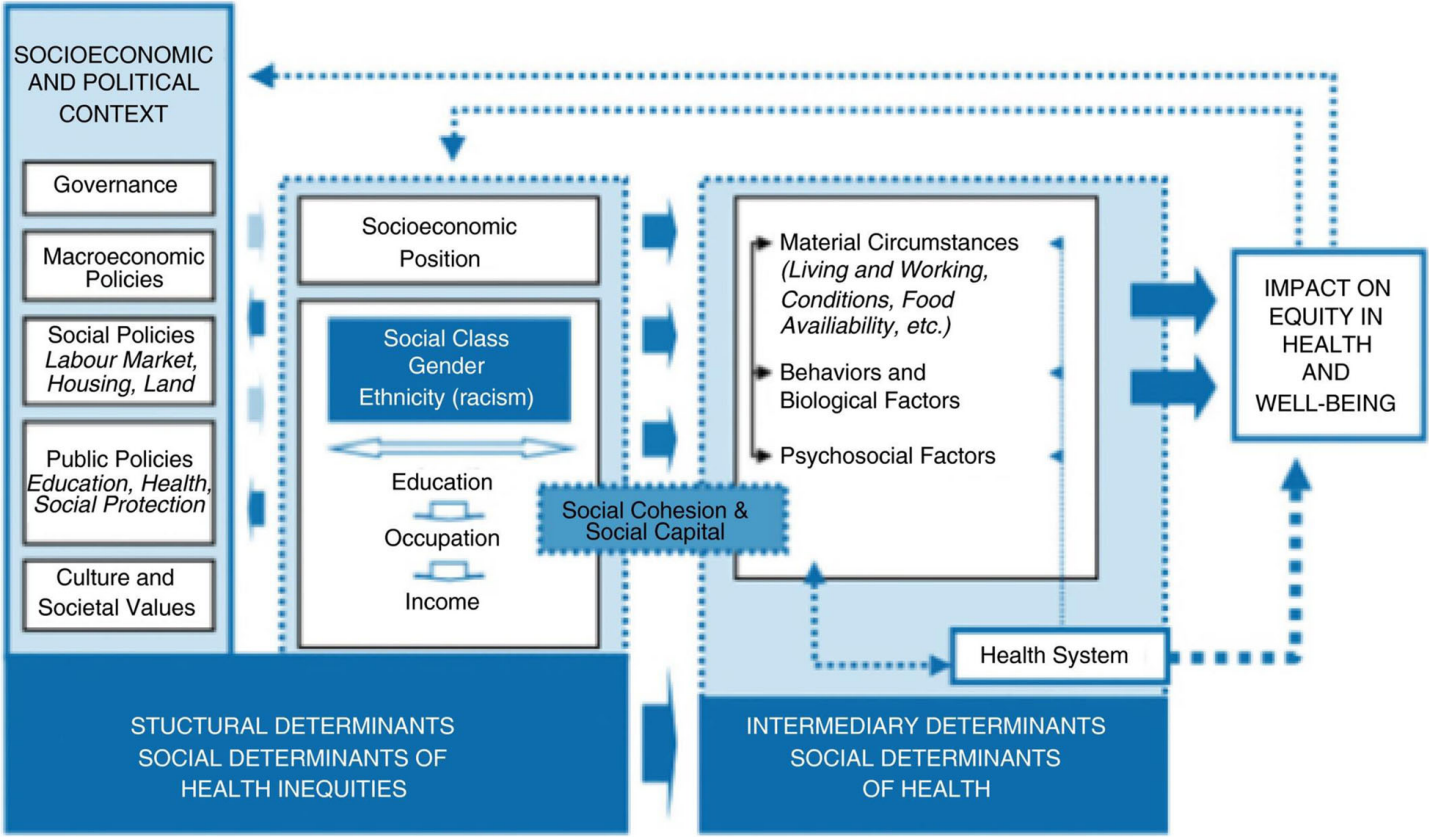
WHO/ CSDH Conceptual Framework [1]

As framework in Fig. 1 illustrates, contextual factors such as social, economic, and political mechanisms play a key role in determining a population’s socio-economic position, which is stratified according to livelihood, income, and education level. These structural determinants then give shape to intermediary determinants, which include living and working conditions, behaviors, biological factors, etc. The intermediary determinants, in tandem with the available health care system, then directly determine health and health outcomes. Due to its proximal relationship to health, health care policy has traditionally directed most of its focus on prevention activities at the intermediary level prior to the onset of poor health, and to treatment after. However, as Fig. 1 demonstrates, this myopic approach fails to address the true root of the problem – the structural inequities that gave rise to such conditions in the first place. Because a person’s health is the result of the complex convergence of multiple factors that are largely out of their control, it becomes apparent that the best way to achieve equitable and sustainable good health is by expanding the focus of healthcare interventions from the individual level to include the system itself (i.e. society).

The WHO SDH framework is a human rights-based model that precisely endorses such an approach. To make this shift, however, it is necessary to first consider the main driver behind how societies evolve: power. According to this framework, those that possess the most power and resources within a given society determine the prevailing priorities, values, and beliefs. In most cases, the decisions that these power wielders make in terms of laws, policies, and other determinants disproportionately benefit themselves and those that share their priorities. Unfortunately, however, these decisions often have negative consequences for marginalized groups - such as indigenous peoples - that hold divergent priorities and values. In contrast with the powerful, such decisions serve only to further constrict their options across various domains such as livelihood, education, and living conditions. The collective and synergistic consequences of these reduced options are often poorer health and health outcomes [1].

The following example illustrates how this interdependent, power-driven process unfolds: at the structural level, a decision is made about labor policy that expands job opportunities for one portion of the population (i.e. those with power), while simultaneously reducing opportunities for another portion with less power. This reduction in job opportunities may then result in lower income potential, which thereby reduces the ability to make choices regarding such things as living conditions and nutrition. With fewer options available, the marginalized population may then have to settle for inferior housing or unhealthy foods, which often results in serious health consequences.

The SDH model is quite logical and provides a very compelling model to explain the large disparity between the health of indigenous populations and others living within the same vicinity. It also provides a clear roadmap as to where health policy and healthcare interventions should be targeted to achieve better outcomes - especially for vulnerable and marginalized populations such as the Urak Lawoi’ and other indigenous groups.

### Background of the UL

The UL are one of three “sea nomad” groups that inhabits Thailand’s southern Andaman coast. Due to the lack of a written language and a reliance on oral tradition, their exact history is unknown. However, linguistic evidence suggests that they originated in Indonesia and gradually migrated to their current location over the past several hundred years. They were first officially recorded in Thailand in 1909 when they played a key role in securing the Adang Archipelago for the kingdom of Siam. For this contribution, the Thai Princess Mother Srinagarindra granted them citizenship in 1968 [5].

The UL are a peaceful, shy, and proud people that traditionally maintained their culture by avoiding contact with outside populations. Their religion is animistic, and they hold a sacred respect for their ancestors; these values are embodied in sacred ceremonies such as the *Loy Rua* celebration, a three-day community-wide celebration that pays homage to UL ancestors, traditions, prayers for the future, and the sea [3, 6]. While most UL continue to take pride in their unique cultural practices and beliefs, their lifestyle is rapidly changing in both positive and negative ways due to globalization, and it is unclear what the future will hold for them.

## Methods

This study sought to identify the SDH that have most impacted the UL in current and recent times. To accomplish this, the researcher conducted an exhaustive review of the most pertinent international mechanisms and national laws and policies. In addition, the researcher also conducted multi-method data collection in three distinct UL communities over a three-month period. The determinants deemed to be most prominent by this mixed-method approach were then analyzed to better understand their interaction, synergy, and impact. The framework below offers a modified, contextualized version of the WHO model (see Fig. 1), and provided the basis for this study’s queries:

**Fig. 2:**
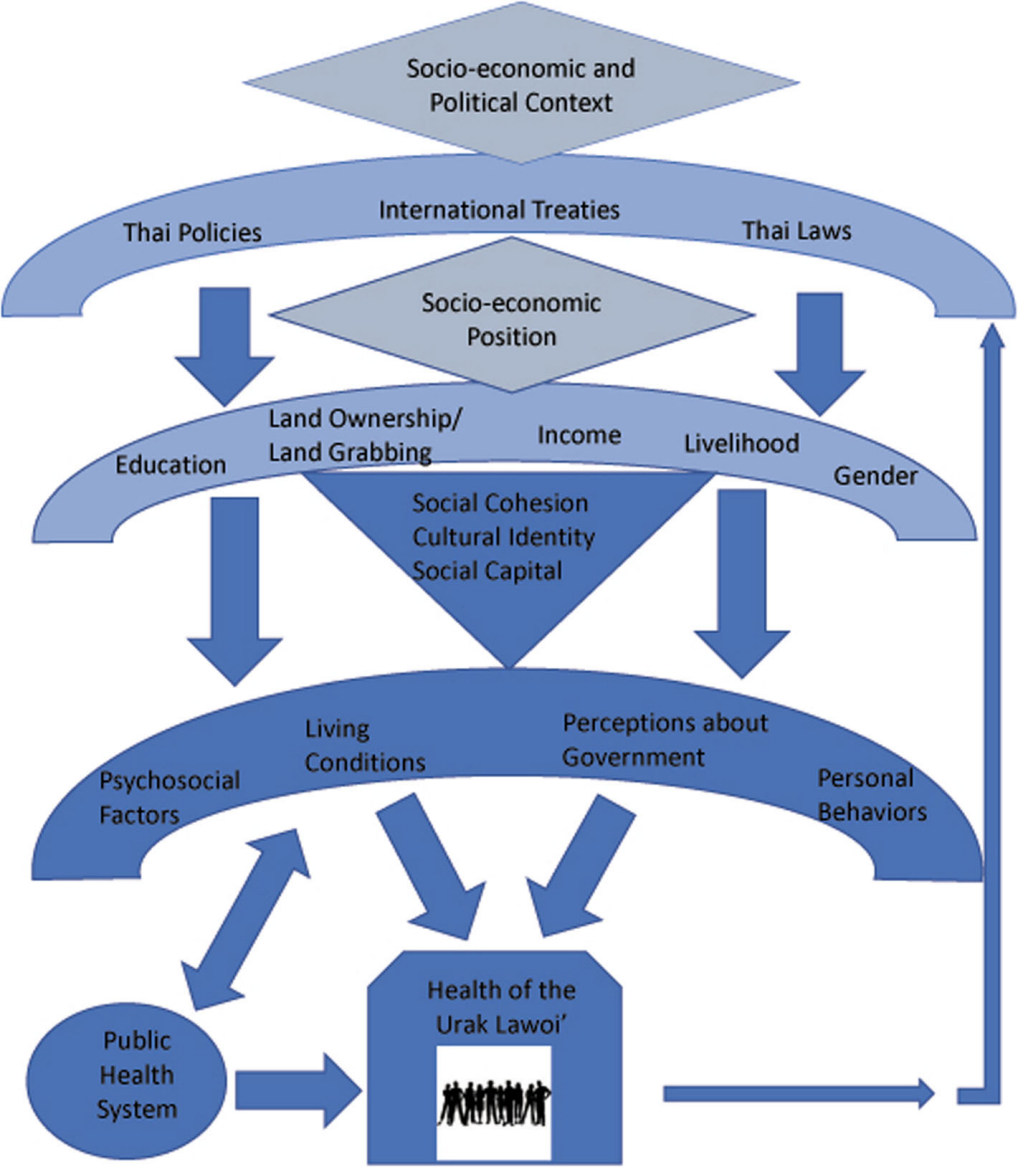
Conceptual Framework & Study Determinants. Derived from WHO Framework [1]

This framework was adapted from the WHO’s original horizontal structure to a tiered vertical structure that better reflects the strongly unidirectional role that the structural determinants play in shaping the health and lives of the UL and other indigenous peoples. The vertical structure more accurately represents the lack of power that has historically characterized the UL people in Thai society. Within the current paradigm, it is quite difficult for the UL to enact the changes needed to dramatically improve their circumstances. Rather, substantial and sustainable improvements are more likely to result from strategic actions taken at the top.

An additional change to the framework can be seen with the feedback loop. The original WHO framework suggests that the structural determinants are subject to change in response to feedback from the intermediary determinants and represents this with thick arrows that point in both directions. In the case of the UL, however, this feedback mechanism is extremely weak, largely due to a lack of power and government representation. Thus, for the purpose of this study, the framework was modified to include a very thin upward arrow, which represents the inferior feedback loop available to the UL, and several wide downward arrows that represent the significantly more powerful influence the structural determinants have on the circumstances of the UL.

A final change to the framework includes the addition of “land ownership/land grabbing” as a stand-alone structural determinant and “perceptions about government” as a stand-alone intermediary determinant. The addition of these determinants was considered necessary because the preliminary literature review revealed that these have been key features in the lives of the UL for at least the past several decades. All the determinants identified in this adapted framework were examined during this study; however, due to word limitations, only the four that were found to be most prominent are discussed in-depth in this article.

### Location and population

This research was completed in three distinct communities on the islands of Ko Lanta and Ko Lipe in southern Thailand. These communities were chosen because they are among the largest known UL communities in Thailand, yet each is unique in terms of resources, remoteness, living conditions, and exposure to other populations.

On Ko Lanta, the communities of Saladan and Sang Ka Ou were studied. Saladan holds a key position at the northern tip of this island, and almost everyone arriving to or departing from the island must pass through here; as a result, the UL living here have frequent interactions with outsiders. There is a government school based here that provides education until the 9th grade, and there is also a centrally located primary healthcare clinic that employs one credentialed UL nurse.

The second community, Sang Ka Ou (~pop. 300–400), is situated on the southeastern tip of Ko Lanta. Due to its remoteness, it has not experienced the same level of development that has transformed the rest of the island, and interactions with outsiders are infrequent. There is a primary school located here that provides education until grade 6, while the high school and nearest healthcare facility are 8 km away.

The final study community is located on the small islands of Ko Lipe and Ko Adang (UL population 1064). These islands are part of the Tarutao Archipelago and situated close to the Malaysian border. The remote location of these islands kept many outsiders at bay until the 1990’s. However, the 2004 tsunami catapulted this island onto the radar of tourists and developers, and it is now a popular vacation destination. As such, over the past 15 years, the UL that live here have had increasingly frequent interactions with the outside world.

### Methodology: data collection

This study utilized a qualitative approach that included data collection and a comprehensive law and policy review. To ensure the validity of the findings, multiple methods of data collection were employed in each of the communities; these included key informant interviews (KII), household surveys (HS), and focus groups (FG). For further triangulation, direct observation and a literature review were also utilized.

Prior to the initiation of the data collection phase, all research instruments were prepared, tested, modified, and re-tested. A pilot test was conducted to test the validity and reliability of the tools. The document review was conducted between February 1, 2016 and December 1, 2016, while data collection was conducted between August 15, 2016 and December 5, 2016. Key informants included community leaders and representatives of the Departments of Education and Health. HS and FG participants included UL men and women that met the inclusion criteria (i.e. over 18 and able to give informed consent). The sample size was based on the principle of saturation, and mixed purposive sampling (criterion sampling and “snowballing”) was used to select the participants.

A Thai - English interpreter was utilized throughout the course of the study. Prior to the initiation of data collection in each of the communities, the researcher and interpreter approached the local *To Maw* and/or assistant chairman to explain the study and its purpose. After rapport had been built and permission granted, these community leaders proved highly instrumental in facilitating data collection by assigning local intermediaries that assisted the research team in recruiting participants and arranging interviews.

In Saladan, 5 KII were conducted with 2 assistant community chairmen, a *To Maw* (medicine man), and a local UL nurse. 15 HS were conducted (5 women, 10 men), and 2 FG (1 gender segregated, 1 mixed) were conducted with 14 participants (10 women, 4 men). In Sang Ka Ou, 2 KIIs were conducted with a *To Maw* and an ethnic Thai principal from the local high school, 10 HS were conducted (8 women, 2 men), and 2 gender segregated FG were conducted with 10 participants (6 women, 4 men). On Ko Lipe, 4 KIIs were conducted with an assistant chairman, an UL schoolteacher, and an ethnic Thai healthcare professional from the local clinic; and 11 HS were conducted (8 women, 3 men). Additionally, 2 HS (1 woman, 1 man) were conducted on the neighboring island of Ko Adang. FG were not conducted on either Ko Lipe or Ko Adang after it became apparent that this was not the best way to collect unique data in this highly communal culture. Table 1 below summarizes the study’s sampling framework and sample size:

**Table 1:** Sampling Framework and Sample Size

HOUSEHOLD SURVEYS				
	*Saladan*	*Sang Ka Ou*	*Ko Lipe and Ko Adang*	*Total*
Women 50+	4	1	2	7
Women 18–49	1	7	7	15
Men 50+	7	0	3	10
Men 18–49	3	2	1	6
	15	10	13	38
FOCUS GROUP DISCUSSSIONS				
	*Saladan*	*Sang Ka Ou*	*Ko Lipe and Ko Adang*	*Total*
Women 50+	0	4	0	4
Women 18–49	10	2	0	12
Men 50+	2	1		3
Men 18–49	2	3		5
	14	10	0	24
KEY INFORMANT INTERVIEWS				
Community elders (*To Maw*), community chairmen, school principal, schoolteacher, healthcare workers, nurse				
	*Saladan*	*Sang Ka Ou*	*Ko Lipe and Ko Adang*	*Total*
	4	2	3	9
		TOTAL PARTICIPANTS		71

Notes and recordings were used to document all participant responses. All collected data was coded and analyzed using a deductive and inductive process, and HyperRESEARCH software was utilized to organize and code all responses.

### Methodology: law and policy review

The study’s document review included an in-depth analysis of several international mechanisms that included the United Nations Declaration on the Rights of Indigenous Peoples (UNDRIP) [7], International Convention on the Elimination of All Forms of Racial Discrimination (CERD) [8], International Convention on Economic, Social and Cultural Rights (ICESCR) [9], Convention on the Rights of the Child (CRC) [10], International Convention on Civil and Political Rights (ICCPR) [11], Convention on the Elimination of All Forms of Discrimination Against Women (CEDAW) [12], and the Convention on Biological Diversity (CBD) [13]. Several Thai laws and policies were also reviewed, including the 2007 Constitution of Thailand [14], Thai healthcare policy [15], the National Education Act of 1999 [16], the National Language Policy (2010) [17], Fisheries Act of 1947 [18], Royal Ordinance on Fisheries (ROF) B.E. 2558 (2015) [19], National Park Act B.E. 2504 (1961) [20], Wildlife Preservation and Protection Act, B.E. 2535 (1992) [21], Land Code of 1954 [22], several current and defunct land reform measures [23, 24], and the Thai Civil and Commercial Code [25].

Regarding international mechanisms, key considerations included how they pertained to the specific circumstances of the UL across various life domains, and what protections they guaranteed. Key considerations for the national law and policy review were the extent to which they complied or deviated from the international mechanisms, as well as the impact that they had upon determinants such as education, health, living conditions, culture, and ethnic identity.

## Results

This study’s findings are summarized below in Table 2. These summaries reflect the recurrent themes that emerged within each community during this study. Themes were deduced when multiple participants reported similar experiences with and/or perceptions about the specified determinant.

**Table 2:** Results

		STUDY COMMUNITIES		
		Saladan	Sang Ka Ou	Ko Lipe
DETERMINANTS AS REPORTED	Livelihoods and Income	Income from traditional livelihoods significantly decreased due to laws, policies, and competition; Limited alternative opportunities, especially for women	Income from traditional livelihoods significantly decreased due to laws, policies, and competition; Limited alternative opportunities, especially for women	Income from traditional livelihoods significantly decreased due to laws, policies, and competition; Limited alternative opportunities, especially for women
	Education	Multiple concerns about curriculum; Limited opportunities to continue education beyond 9th grade	Multiple concerns about curriculum; Limited opportunities to continue education beyond 9th grade	General satisfaction with curriculum, but would like more English training; Extremely limited opportunities to continue education beyond 9th grade
	Land Ownership and Land Grabbing	Land is government owned; Access to ancestral burial grounds in vicinity is at risk	Most participants own their land and homes and are relatively satisfied; Some participants reported that they were coerced into moving away from the sea in exchange for aid	Land grabbing is a significant issue for entire community, with imminent threat of eviction for at least 121 households; A portion of ancestral burial ground has been claimed by nearby resort
	Living Conditions	Substandard living conditions in Toh Ba Lue include: Poor access to running water; Poor sewage and sanitation, Poor and unsafe access to electricity; Interior of many homes exposed to the elements and biting insects	Limited water supply during dry season; No trash removal services	Substandard living conditions for several homes; Poor access to running water

The study examined a total of eleven determinants. Due to article word limitations, only the four most significant determinants are discussed here in greater depth. The most prominent themes that emerged during the analysis were “insufficient livelihoods and income-generating potential”, “lack of education”, “land insecurity and land grabbing”, and “inferior living conditions”. Within each of these themes, various sub-themes emerged to different degrees across the three study communities. Regarding “insufficient livelihoods and income-generating potential”, the prominent sub-themes were 1.) decreased ability of UL to earn sufficient income from their traditional livelihood as fishermen, and 2.) low availability of adequate and consistent alternative livelihood options.

The second prominent theme that was identified was “lack of education”. Sub-themes that emerged here were 1.) inaccessibility, and 2.) lack of a relevant curriculum. The third most prominent theme to emerge was “land insecurity and land grabbing”; this theme was characterized by the sub-themes of 1.) overly complex bureaucratic processes, and 2.) corruption. The fourth most prominent theme was “living conditions”, which was characterized by the sub-themes of 1.) substandard facilities, and 2.) limited ability to make necessary repairs.

Each of the above-mentioned themes are significant determinants of health. Taken individually, they place the UL at greater risk of poorer health and health outcomes, and this becomes increasingly true when the determinants synergize. In the discussion section, we shall explore in greater depth each of these themes and sub-themes, their synergies, and their potential impact on health.

## Discussion

### Insufficient livelihoods and income-generating potential

The most pervasive and recurrent theme that emerged in all three study communities was that of livelihoods and income-generating potential. Across all three communities, almost all participants expressed concerns about their ability to make ends meet in their traditional roles as fishermen. Many reminisced about the past when they could fish wherever they wanted while utilizing their traditional and largely sustainable fishing techniques. In those days, they could catch enough fish to feed their families, and then share the remainder with others in the community. This has changed dramatically over the past several decades; nowadays, fishermen bring home much smaller yields from the sea, and this severely impacts their ability to earn a decent living and put food on the table just for their own households.

Unable to make ends meet, many UL men and women have begun working outside their communities in non-traditional roles. However, most of the positions available to them are low paying seasonal jobs in tourism and construction. Better jobs with higher wages are largely inaccessible to the UL because most lack sufficient formal education, external social capital, and power. As the cost of living rapidly increases in their island communities, most are now surviving barely at a level of subsistence.

Referring to Fig. 2, livelihoods and income fall into the second tier of structural determinants which, according to the WHO, are largely influenced by top-tier structural mechanisms. The document review portion of this study reveals that this is indeed the case here, with laws and policies playing a major role in several ways. Thailand has signed several international treaties that guarantee the rights of all individuals to pursue the livelihood of their choice, including the ICESCR [9], ICCPR [11], and CEDAW [12]. Moreover, the 2007 Thai Constitution [14] also guaranteed this right. However, national laws such as the 1947 Fisheries Act [18], the 2015 Royal Ordinance on Fisheries [19], the 1961 National Park Act [20], and the 1992 Wildlife Preservation and Protection Act [21] have imposed numerous restrictions on the movements and activities of UL, and many of their ancestral fishing grounds are now off limits. Although the UNDRIP [7] guarantees additional livelihood protections to indigenous groups, this does not benefit the UL as Thailand refuses to recognize them as indigenous. Current policy confines them to the same fisheries used by large-scale commercial fishermen with whom they simply cannot compete. These commercial fishermen not only over-fish the waters, but they also frequently destroy UL fishing equipment during their large trawling operations.

Referring again to Fig. 2, the trickle-down effects of the structural determinants found in the first and second tier then manifest themselves in the intermediary determinants located in the framework's third tier. Insufficient income limits the ability of many UL to obtain nutritious foods, which can lead to malnutrition [26]; potable water, which increases the risk of waterborne and parasitic diseases [27]; and medicines, which can result in acute or chronic illnesses. Furthermore, inadequate income may also cause some UL to resort to dangerous work - such as deep diving and construction – as a means of paying for necessities; this substantially increases the risk of injuries.

### Lack of education

Access to education – a second-tier structural determinant (see Fig. 2) - is also an important SDH because it is closely linked to the type, quality, quantity, and availability of livelihood opportunities, as well as the ability to make informed and healthy behavioral choices. In all three communities, it was found that most young people drop out of school before completing the 12th grade and therefore lack a high school diploma. Moreover, very few of those who do manage to graduate continue to pursue any sort of higher education. The primary reasons given for this were dissatisfaction with the available curriculum, extremely limited access to higher education opportunities, and economic pressure to help their families by making money.

Again, it is clear that these are strongly influenced by the top-tier structural mechanisms seen in Fig. 2. Although Thailand’s 2007 Constitution [14] and current education policy [16] guarantees 12 years of education to all citizens, the education system tragically fails in terms of ensuring accessibility to this population. For all three UL communities, the costs of continuing to secondary school are prohibitive. This is a particularly significant challenge on Ko Lipe, with the nearest secondary school over 60 km away by sea. The dream of attending university is even more elusive for these same reasons. This ubiquitous lack of education severely limits livelihood options and confines many UL to a cycle of poverty.

In addition to accessibility challenges, the high dropout rate is also not surprising given the pervasive dissatisfaction with the curriculum; over the course of this study, only a few UL described the available curriculum as beneficial. In all three communities, participants expressed the need for improved and expanded English language instruction, which they believed would significantly enhance their career opportunities. Thailand’s education policy regarding language currently prioritizes English as a second language [17], and many schools on the mainland have taken steps to enhance the quality of their English programs. However, the English lessons offered in the three study communities remain rudimentary and insufficient to support proficiency.

Finally, in Saladan and Sang Ka Ou, the curriculum lacks cultural relevance for the UL. In these two communities, many participants identified the loss of important cultural features - such as the UL language - as a significant threat to their community, with many young people now only able to speak Thai. The CRC [10] guarantees the right for children to “learn about and practice their own culture, language and religion”, while the UNDRIP [7] obliges states to implement measures to protect the culture, language, and way of life of indigenous peoples. The local schools should be venues where Thailand honors these commitments; this would also be in compliance with the country’s current education policy, as the National Education Act of 1999 [16] mandates a standardized curriculum that reflects national priorities but is also adapted to meet unique community needs. No one should be forced to speak a language that they do not wish to speak; however, for the UL in these communities, the choice is gradually being taken from them due to a rigid education system that fails to embrace their distinct cultural identity. Here, the inadequate feedback loop identified in Fig. 2 perpetuates the current paradigm. The UL lack adequate representation in the education system and are therefore unable to influence the type of education available to them; thus, they place a low priority on a formal education that they perceive to be irrelevant. If this circular process remains unchanged, the UL as a people will continue to lack the power needed to influence the nature and quality of the education available to them.

In terms of cultural relevance, an exception to the above was noted at the school on Ko Lipe, which has established itself as a progressive model offering a bilingual, bicultural curriculum that celebrates the UL identity. Notably, participants on Ko Lipe expressed the most satisfaction with their school, and they also appeared to have retained the strongest cultural identity of the three study communities. If the schools in Sang Ka Ou and Saladan followed suit and incorporated at least some degree of bilingual, bicultural education into their curriculum, they could potentially slow cultural erosion, support social cohesion, and increase school attendance.

The lack of education impacts intermediary determinants such as food availability, behaviors, and materials circumstances (see Fig. 2), and this is correlated with increased health risks such as decreased fertility rates [28] and higher incidence of conditions such as hypertension, cardiovascular disease, stroke, and diabetes mellitus [29]. While these non-communicable diseases are largely due to personal behaviors, less education means less information is available to make informed and healthier behavioral choices, such as limiting salt intake, increasing physical exercise, and eliminating smoking [30]. Moreover, the costs of treating such chronic diseases are exorbitant and place a severe strain on national and local economies. Funding deficiencies may then result in the reduced availability of public healthcare services, which further compromises the health of marginalized indigenous groups such as the UL [31].

### Land insecurity and land grabbing

Although not specifically mentioned in the WHO framework [1], an initial review of the literature suggested that land insecurity and land grabbing have significantly impacted the lives of the UL; thus, these determinants were included in the modified framework presented in Fig. 2. These determinants were found to be particularly relevant on Ko Lipe, where the on-going fear of eviction is a stressor that impacts every domain of their lives. Although the top-tier structural mechanisms of laws and policies should have ensured land security, bureaucratic complexities in tandem with systemic corruption has resulted in the ongoing loss of UL land holdings. When the Thai government granted all UL citizenship in 1968, they gained the same rights as other Thai citizens, including the right to own land. Per Thailand’s Civil and Commercial Code [25], most of the UL that lived on Ko Lipe at that time were eligible to legitimately claim ownership of the land they occupied, and Thailand’s Land Code [22] details the required registration process. Regrettably, however, the process is neither easy nor intuitive.

In 1968, most UL were illiterate and incapable of navigating the complex land registration process. They also did not fully comprehend the concept of land ownership, which was quite foreign to their traditional and communal way of life. The Thai government failed to provide them with the assistance that they needed to complete this process, and corrupt officials exploited their lack of power and legal naïveté to steal their land. This dire situation continues today, and little of substance has yet been done to rectify these wrongs. If a solution is not arrived at soon, most of the UL currently living on Ko Lipe may be completely and permanently evicted from the very island that their ancestors helped secure for Thailand over a century ago. This uncertain land tenure reduces the ability of the UL to make necessary modifications or repairs that improve their living conditions, and this substantially increases health risks.

Land grabbing is also threatening the ability of UL to utilize and visit their ancestral cemeteries, and they lack the power to successfully challenge this paradigm. This is true both on both Ko Lipe and Ko Lanta, where many developers purchased land through questionable transactions, and then restricted access to the UL’s traditional burial grounds. In some circumstances, graves were desecrated and human remains illegally exhumed. This is a blatant violation of the UNDRIP [7], which guarantees that indigenous peoples have the right to “maintain, protect, and have access in privacy to their religious and cultural sites” and the “right to the repatriation of their human remains”.

Referring to Fig. 2, here we again see the trickle-down impact of the above mentioned first- and second-tier structural determinants on third-tier intermediary determinants. On Ko Lipe, the ongoing displacement has heavily influenced perceptions about the government; here, many UL described their village headman as corrupt and expressed feelings of betrayal and abandonment by the Thai police and government. Chronic perceptions of distrust and discrimination can lead to chronic stress and anxiety - both of which have been linked to coronary heart disease and hypertension [32]. Distrust can also reduce the likelihood that individuals will seek and comply with healthcare directives [33], and it may also increase the risk of engaging in harmful coping behaviors such as using tobacco and abusing alcohol [34].

### Inferior living conditions

As per Fig. 2, living conditions is a third-tier intermediary determinant that can also significantly impact health, and it was repeatedly identified as a prominent concern by numerous UL households. This determinant is largely influenced by the previously discussed second-tier structural determinants of livelihoods and income. However, the literature review revealed that this is also influenced by additional top-tier laws and policies, and therefore warrants further examination and discussion.

Almost all UL living in the Toh Ba Lue neighborhood of Saladan, and many of those living on Ko Lipe, are living in substandard, dilapidated housing that lacks basic plumbing and other utilities. Because many of the UL living in these communities are considered “squatters”, legal restrictions prevent them from repairing or upgrading their homes. Many also lack the financial means needed to make essential repairs, while others hesitate to invest money in a home from which they fear they will eventually be evicted. Although the 2007 Constitution [14] promises suitable living conditions to all Thai citizens, the government has so far failed to provide adequate assistance to the UL. Moreover, the weak feedback mechanisms illustrated in Fig. 2 deny them the power to negotiate this. Thus, for many UL households, the possibility of improving living conditions to meet minimum standards remains elusive.

On Ko Lipe, the ongoing land ownership struggle was the biggest factor impacting living conditions. Several of the homes visited during this study lacked plumbing, electricity, and other utilities, and some required extensive structural repairs. Here, land ownership battles also hampered the ability to make necessary improvements. According to Krieger and Higgins [35], individuals living in substandard conditions such as these are more prone to infectious diseases and susceptible to mental health problems, and they also face increased risk of exposure to harmful vermin and insects that serve as vectors for numerous communicable diseases.

### Synergies

Although the discussion thus far has presented the various determinants as independently operating determinants, it is important to emphasize that there is significant interdependence between the various determinants. While the framework presented in Fig. 2 alludes to this interdependence, the case of the UL provides a clear demonstration of how this works. As we have seen, “insufficient livelihoods and income-generating potential” was a recurrent theme in all three of the study communities, with many UL households now seeking alternative sources of employment to make ends meet. However, their options for stable and profitable employment are extremely limited due to the pervasive “lack of education”. The lack of formal education limits most UL to low-paid labor jobs in construction or tourism, which restricts their ability to pay for necessary improvements in their living conditions. It also impedes the ability of many UL to navigate highly complex bureaucratic processes and leaves them highly vulnerable to exploitation by corrupt officials and land developers; this increases the risk of “land insecurity and land grabbing”. This, in turn, further restricts their ability to address “inferior living conditions”, and effectively deprives them of access to proper sewage, sanitation, electricity, and clean drinking water. Meanwhile, the lack of power, representation and other feedback mechanisms severely restricts the ability of the UL to positively change any of these determinants. Thus, if the current paradigm continues, many UL will remain trapped in a complex web of circumstances that places them at increased risk of poorer health and health outcomes than their ethnic Thai neighbors.

## Conclusions

Consistent with the WHO framework presented in Fig. 1, the evidence gathered through this study strongly suggests that the UL - like many indigenous groups around the world - are impacted by several interdependent SDH that place them at increased risk for poorer health and health outcomes. At the structural level, laws and policies have been established that benefit mainstream Thai society and those in power; however, these largely fail to consider or meet the needs of the UL, and the UL lack the representation and other feedback mechanisms needed to change this. Several maritime laws and conservation acts that directly threaten the primary livelihood of the UL have been implemented over the past several decades. Few steps have been taken to fill the resulting livelihood gap, and many UL are now forced into menial jobs that barely allow them to meet basic subsistence needs. Limitations in accessibility to quality education further reduce livelihood options. Moreover, education policy actively pursues a path of assimilation that fails to embrace the ULs unique cultural identity, thus contributing to low attendance rates and cultural erosion.

For the UL in the study communities, land ownership and land grabbing are particularly critical, especially on Ko Lipe. While existing Thai policy and laws should protect land rights, many UL are unaware of their rights due to illiteracy and lack of education. Thus far, the Royal Government of Thailand (RTG) has not provided adequate assistance to help them understand and exercise their rights. Despite the critical role that the UL played in securing the Adang Archipelago for the Kingdom of Siam in 1909, their land is being appropriated at alarming rates, and there is nowhere else for them to go.

Thailand has signed numerous international treaties intended to protect human rights by guaranteeing equitable access to quality education, healthcare, and protection of ancestral lands and cultural identity. The most recent Thai constitutions have also ensured these rights; however, existing laws and policies largely fail to fulfill these obligations. Without immediate and effective interventions from the RTG, civil society, and other stakeholders to change this paradigm, the traditions, lifestyle, livelihood, land, health, wellbeing, and very existence of the UL are at imminent risk.

Proponents of the SDH model [36] argue that health inequities are best addressed when governments make appropriate top-tier structural changes such as amending policies and laws. To do so effectively, it is critical to strengthen feedback loops by including the UL communities in making decisions about the laws and policies that impact them. The RTG can accomplish this by taking steps to ensure that the UL have adequate representation at the national, provincial, and local levels of government; this would substantially reduce inequities and increase their power.

Such an approach was utilized with the indigenous Māori of New Zealand, who were granted four dedicated Parliamentary seats in 1867; in 2002, this number was increased to seven. The allocation of seats not only increased the Māori’s power to directly weigh in on policies and laws that impact them, but it also helped stem intergroup tensions. Today, compared to other indigenous groups, the Māori maintain a relatively powerful position in their government and society [37]. Although the UL have a substantially smaller population than the Māori, this affirmative action offers an example of how Thailand can ensure that they (and other indigenous groups within its borders) are included in relevant decision-making processes. In the next section, several additional recommendations - based on WHO guidance - are offered.

### Recommendations

Per the SDH framework presented in Fig. 1, governments must play a primary role in ensuring health equity for all by ensuring that services such as education and health, and resources such as water, sanitation, and nutritious foods are available on an equitable basis. To achieve this, governments should implement and enforce inclusive multi-sectorial policies and laws at both the national and local level. These laws and policies should be complementary; if not, there is a significant risk that they may worsen health conditions and increase health disparities for the most vulnerable [38].

In the case of the UL, the undisputedly most significant step that the RTG could take to ensure equity is to recognize them as an indigenous people, and then grant them the rights and protections guaranteed by the UNDRIP. By so doing, the roadmap moving forward would be clearly defined, with additional implementation guidance available from the United Nations Development Group (UNDG) [39]. However, as Thailand has so far refused to recognize any indigenous groups within its borders, this is unlikely to change in the near future. Regardless, the RTG can and should still enact and enforce law and policy changes on an ad hoc basis that would greatly improve the health, well-being, and socio-economic status of the UL and other indigenous groups.

These recommended actions are:

1. Take steps to protect fishing as a traditional livelihood for the UL and support decent alternative livelihood options. This could include exempting them from maritime laws and conservation measures that restrict their ability to utilize their traditional fishing techniques in their ancestral fisheries, and/or helping them develop safe and profitable alternative livelihood opportunities.
2. Ensure that public education is culturally relevant and better suits their livelihood needs (i.e. better English training and/or suitable vocational training), and higher education is more accessible; this would enable the UL as a people to increase their power and external social capital. This could be accomplished by providing the UL with financial assistance, scholarships, and/or other incentives. The RTG can also encourage schools in UL communities to include a bicultural, bilingual component that celebrates the unique cultural identity of the UL. Finally, the RTG should take steps to ensure that the education provided in UL communities matches their priorities, such as an improved and expanded English curriculum, which would then enable them to find better jobs in the tourism sector.
3. Take steps to rectify past land grabbing abuse by ensuring land claim investigations and proceedings are unbiased, transparent, and free of corruption. Additionally, take steps to prevent any future abuses by consistently and fairly enforcing prescription and inheritance laws. The RTG should also assist those UL who are landless and without remedy by establishing permanent reservations in culturally appropriate locations where they can live in peace without fear of eviction. Given the power deficits of the UL relative to other Thais, the RTG should explore strategies to ensure that UL voices are heard, and their interests equally considered, in all land dispute decisions that directly or indirectly affect them. Such strategies should include ensuring that UL communities are aware of relevant competing land claims as they emerge, and they have adequate legal representation during judicial proceedings regarding land to which they claim ownership. Finally, the RTG should establish easements so that the UL can more easily access their ancestral cemeteries from which they have been cut off due to past land development.
4. Take steps to increase opportunities for participation by UL on matters that directly or indirectly affect them; this can be achieved by providing venues where the UL can freely voice their opinions, and by ensuring that they have consistent representation at the local, regional, and national level.
5. Adopt and enact the guidelines proposed by the UNDG [39] by collecting and maintaining ethnically disaggregated statistics reflecting ethnicity and regarding epidemiology, healthcare utilization, education, livelihood, income, and representation in government.

While this researcher offers these recommendations to the RTG as a first step they can take to reduce the health inequities faced by the UL, it is critical for civil society to also act. At the intermediary level, civil society should work closely with UL communities to design and implement initiatives that address inequities and increase their power. At the structural level, civil society should advocate and hold the RTG accountable. As a final recommendation, civil society should also join with academia to conduct additional research and document injustices and inequities that negatively impact the health and well-being of the UL and other indigenous groups in Thailand.

### Limitations

For this study, the following potential limitations were identified: 1.) Due to logistical challenges, four different interpreters were utilized over the course of the study, and it is feasible that interpretation was not applied in a consistent manner. Eventually, a professional interpreter from outside the region was employed. Her young age (27 yo) and the fact that she was an outsider may have potentially impacted participant responses; 2.) Due to the sensitive nature of some of the questions, it is feasible that information may have been underreported or exaggerated. Moreover, the presence of a local community intermediary, who was needed to gain access to this somewhat xenophobic culture, may also have affected responses; 3.) Although all participants spoke Thai, it is their second language; it is therefore conceivable that some research questions may have been misunderstood; 4.) It was not possible to consider all SDH (i.e. economic policy, genetics, nutrition, etc.); thus, it is feasible that other critical determinants were overlooked; 5.) Health statistics disaggregated by ethnicity do not exist in Thailand; thus, it was not possible to specifically identify existing health disparities; and 6.) This research focused on one indigenous group living in three different island communities; it is quite feasible that circumstances in other UL communities are significantly different.

## Data Availability

The datasets used and analyzed during the current study are available from the corresponding author on reasonable request.

## Abbreviations

CBD: Convention on Biological Diversity
CEDAW: Convention on the Elimination of All Forms of Discrimination Against Women
CERD: International Convention on the Elimination of All Forms of Racial Discrimination
CRC: Convention on the Rights of the Child
FG: Focus groups
HS: Household surveys
ICCPR: International Convention on Civil and Political Rights
ICESCR: International Convention on Economic Social and Cultural Rights
KII: Key informant interviews
RTG: Royal Government of Thailand
SDH: Social Determinants of Health
UL: Urak Lawoi’
UN: United Nations
UNDG: United Nations Development Group
UNDRIP: United Nations Declaration on the Rights of Indigenous Peoples
WHO: World Health Organization
